# Isolation and Validation of Host-Derived Probiotics From the Giant Freshwater Prawn (*Macrobrachium rosenbergii*): Impacts on Water Quality and Growth Performance

**DOI:** 10.1155/anu/9156848

**Published:** 2025-11-26

**Authors:** Md. Adil Mahfuz, Tamanna Arefin Nobony, Abdul Kader Jilani, Abir Hasan, Md. Nazmul Islam Rifat, Md. Nurul Haider

**Affiliations:** Department of Fisheries Technology, Bangladesh Agricultural University, Mymensingh, Bangladesh

**Keywords:** aquaculture sustainability, growth performance, host-derived bacteria, *Macrobrachium rosenbergii*, probiotics, water quality

## Abstract

Host-derived probiotics offer sustainable alternatives to the commercial nonaquatic formulations due to superior compatibility with the gastrointestinal environment of aquatic species. This study integrated: (i) isolation, identification (via 16S rRNA sequencing), and in vitro potentiality (pH and bile tolerance) of gut-derived bacteria from wild *M. rosenbergii*, and (ii) an in vivo 120-day aquarium trial to evaluate performance of these laboratory-isolated probiotics (T1) compared to a commercial probiotic formulation (T2), and a control group fed with no probiotics (T0). Nine bacterial isolates were identified, including *Bacillus cereus*, *Enterococcus faecium*, *Glutamicibacter mysorens*, and *Staphylococcus succinus*, which exhibited strong acid and bile tolerance. In vivo, probiotic treatments improved water quality by reducing ammonia and stabilizing pH, ammonia was significantly lower in T1 (0.28 ± 0.03 mg/L) than in T0 (0.36 ± 0.06 mg/L; *p* < 0.05). Growth performance was enhanced with probiotics: final average weights reached 3.92 ± 0.08 g in T1, 3.17 ± 0.06 g in T2, and 2.31 ± 0.13 g in T0. Specific growth rate (SGR) was significantly higher in T1 (2.87% ± 0.03%) compared to T0 (2.36% ± 0.05%). Feed conversion ratio (FCR) was lowest in T1 (0.32 ± 0.09) and highest in T0 (1.13 ± 0.14), while feed conversion efficiency (FCE) was highest in T1 (3.15 ± 0.50). Overall, laboratory-isolated/host-derived probiotics outperformed the commercial formulations by simultaneously enhancing growth, feed utilization, and water quality, emphasizing their usefulness as a species-specific, sustainable alternative for freshwater prawn aquaculture.

## 1. Introduction

Aquaculture faces increasing challenges related to disease outbreaks, water quality deterioration, and the overuse of antibiotics. Probiotics—defined as live microorganisms conferring health benefits to the host when administered in adequate amounts [1, 2]—have emerged as promising alternatives in aquaculture systems [3]. They can suppress pathogens [4], enhance innate immunity [5], and improve water quality by reducing organic loads and nitrogenous waste [6, 7].

*Macrobrachium rosenbergii*, the giant freshwater prawn, is a high-value species in South and Southeast Asia, contributing significantly to export earnings and rural livelihoods in Bangladesh [8–10]. However, culture intensification has increased vulnerability to stress and disease [11]. Previous studies reported that probiotics can improve digestive enzyme activity, immune responses, and survival in *M. rosenbergii* juveniles, as well as reduce the risk of disease outbreaks by reducing pathogens and improving water quality [12, 13]. These results highlight probiotics as practical alternatives to antibiotics and chemotherapeutics, which pose significant health risks for farmed prawns and human beings [14].

Probiotics represent a potentially practical approach to address the challenges associated with *M. rosenbergii* culture, and controlled use of probiotics can reduce production costs and increase farmers' benefits. However, commercial probiotics used in aquaculture are typically based on terrestrial or nonnative strains, raising concerns about their adaptability and long-term effectiveness in aquatic environments [15]. In contrast, host-derived probiotics—isolated directly from the gastrointestinal tract of the target species—are expected to colonize more effectively and provide superior probiotic functions due to their adaptation to the host gut environment [16, 17]. However, systematic validation of host-derived probiotics in *M. rosenbergii* aquaculture remains limited.

To address this gap, the present study combined (i) in vitro isolation, molecular identification, and functional screening of gut-derived bacteria from wild *M. rosenbergii* and (ii) an in vivo aquarium trial comparing a host-derived probiotic consortium against a commercial formulation. The dual approach allowed us to establish both the physiological robustness of candidate strains and their practical value in growth, feed utilization, and water quality regulation, thereby providing strong evidence for the development of species-specific probiotics tailored to freshwater prawn culture.

## 2. Materials and Methods

This work comprised two phases. Phase I covered field sampling, isolation, identification, and in vitro tolerance assays; Phase II consisted of an in vivo aquarium feeding trial contrasting lab-isolated and commercial probiotics.

### 2.1. Phase I: Isolation, Identification, and In Vitro Tolerance Assays

#### 2.1.1. Study Area and Laboratory Setup

The study area for this experiment included three upazilas: Kuliarchar (study area-1) of Kishoreganj, Mohanganj (study area-2), and Purbadhala (study area-3) of Netrokona district in Bangladesh. The study region was carefully chosen to facilitate the collection of wild *M. rosenbergii* species from the waterbodies adjacent to the respective areas [8]. The laboratory procedures for this study were carried out in the Fisheries Microbiology Laboratory, Department of Fisheries Technology, Faculty of Fisheries, Bangladesh Agricultural University (BAU) in Mymensingh.

#### 2.1.2. Isolation and Characterization of Bacterial Strains From the Gut of Wild *M. rosenbergii*

For the isolation and characterization of gut-derived probiotic bacterial strains, Lactobacillus MRS (De Man Rogosa, and Sharpe) Agar [18] was primarily used due to its well-known acceptance as a selective medium for culturing lactic acid-producing bacteria (LAB), having a pH of 5.7–6.2. Its nutritional composition and acidic environment are optimized to support the growth and enumeration of LAB effectively [19]. In addition to MRS agar, nutrient agar medium was employed during the isolation process to broaden the diversity of bacterial strains. Additionally, MRS and nutrient broths were used to enrich dissected gut samples, which were then subsequently plated to MRS agar. The detailed protocol is described below.

*M. rosenbergii* were collected from the above-mentioned study areas and dissected to extract their gastrointestinal tracts. Prior to dissection, all *M. rosenbergii* specimens were carefully rinsed with physiological saline to prevent surface contamination. The whole dissection process was conducted on a sterile stainless steel surface. Gastrointestinal tracts were carefully removed using a sterile needle [20].

Sterilized swab sticks were employed to collect samples from the gastrointestinal tracts, which were subsequently transferred meticulously onto preprepared MRS and nutrient agar plates. The collected gut samples and swab sticks were also transferred in Falcon tubes with MRS and Nutrient broth media for enrichment. After incubating the broths at 37°C for 2 h, inocula were again streaked onto MRS and nutrient agar plates. All agar plates were then incubated at 37°C for 48 h. Bacterial colonies obtained from the cultured MRS and nutrient agar plates from the study area were further streaked to get a single colony with particular characteristics [21, 22].

To select a bacterial strain, colony characteristics such as colony size, shape, pigments, and colony margins were examined. Among the colonies having similar characteristics, one representative was considered, and thus, nine isolates were selected for molecular identification as well as a biochemical (pH and bile tolerance test) potentiality test.

#### 2.1.3. Molecular Identification of Isolated Strains

Molecular identification occurred by focusing on the 16S rRNA gene analysis. The 16S rRNA method is commonly employed in the field of microbiology to analyze bacterial classifications. This approach includes DNA extraction from the selected bacterial strains, followed by amplification of the 16S rRNA gene through the polymerase chain reaction (PCR) process with the help of universal primers. The PCR products were then subjected to agarose gel electrophoresis to confirm amplification and subsequently sequenced using the Sanger sequencing method.

#### 2.1.4. DNA Extraction, PCR Amplification, and Agarose Gel Electrophoresis Protocol

The phenol-chloroform method was employed for DNA extraction with minor modifications [23]. A set of universal primers (27F: AGAGTTTGATCATGGCTCAG and 1492R: TACGGTTACCTTGTTACGACTT; [24]) was used to amplify the 16S rRNA gene [25]. The reaction volume of the PCRs was 25 μL, containing 10 ng of template DNA, 2 mM MgCl2, 1 mL of each primer (10 mM), and 1 × Taq Master Mix. The PCR program started with an initial denaturation at 95°C for 5 min, then 35 cycles of 94°C for 30 s, 54°C for 30 s, and 72°C for 45 s, finishing with a final extension at 72°C for 10 min. The PCR products were visualized using a gel documentation system (Bio-Rad). A 1 Kb plus DNA ladder (New England Biolabs, UK) was used to compare the band on an agarose gel. The PCR products were confirmed on agarose gels (1%) stained with ethidium bromide on the gel documentation system (EZEE Clearview UV transilluminator).

#### 2.1.5. Sequencing Methodology

PCR products were purified, and then single-stranded products were generated using cycle sequencing PCR with forward or reverse primers. According to the manufacturer's protocol, PCR products were run on a Sanger machine using the dideoxy chain termination method at Wuhan Tianyi Huayu Gene Technology Co., Ltd.

#### 2.1.6. Analysis of Sequencing Data

The results were processed using specialized software called “BioEdit” [26]. The forward and converted reverse bases were merged to obtain the final base pair. These processed base pairs were input into BLAST (Basic Local Alignment Search Tool) for identification against the NCBI (National Center for Biotechnology Information) database, to identify the closest match or best similarity with the sequences obtained from the study. The highest degree of alignment between the obtained sequence and the sequence stored in the NCBI database is considered the expected species. Multiple sequence alignment was carried out with the CLUSTALW program. The phylogenetic tree has been constructed using the software “Mega 11” [27] based on maximum likelihood.

#### 2.1.7. Biochemical Profiling Based on Potentiality Tests

pH and bile tolerance tests were conducted to assess the potentiality of isolated bacterial strains. Potential probiotic bacteria are capable of surviving in extreme conditions that most bacteria cannot endure. They are naturally found in the gut content and expected to exhibit resilience in extreme conditions similar to the gut environment, such as low pH levels and high concentrations of gastric juice, which include bile salts [28, 29].

##### 2.1.7.1. pH Tolerance Test

The pH tolerance test was conducted by exposing bacterial isolates to different low pH levels (pH 2, 3, and 4). Initially, each isolates were cultured on MRS agar to obtain fresh colonies. A single colony from each isolate was suspended to MRS broth for 24 h at 37°C to reach the logarithmic phase, which typically occurs around 24 h under suitable conditions. The resulting broth culture was diluted with physiological saline (0.9% sodium chloride solution) to prepare uniform bacterial suspensions. The pH levels were adjusted to 2.0, 3.0, and 4.0 using hydrochloric acid or sodium hydroxide. The cell suspensions of the isolates were then exposed to the corresponding pH levels for varying time intervals: 0, 30, 60, and 90 min, plated on Lactobacillus MRS Agar plates, and incubated at 37°C [28–30].

For control, bacterial cells were suspended only in physiological saline without any exposure to low pH and were similarly plated on MRS agar. The growth and colony formation were observed; colonies were counted and calculated according to the following:

  $$\begin{array}{c} \text{Viable count of bacteria in water }\left(\text{CFU},\text{ colony forming unit}/\text{mL}\right)=\left(\text{no}.\text{of colonies on agar plate}\times \text{dilution factor}\right)/\text{volume of culture plated in mL}. \end{array}$$

##### 2.1.7.2. Bile Salt Tolerance Test

Bile salts were dissolved in a sterile sodium chloride solution to prepare bile salt at various concentrations (0.3%, 0.5%, and 1.0%). Before exposing isolates to different bile salt concentrations, bacterial isolates were similarly cultured on MRS agar and then a single colony transferred to MRS broth for 24 h at 37°C. The resulting broth culture was diluted with sterile physiological saline solution to prepare uniform bacterial suspension. The cell suspensions of the isolates were then mixed with the corresponding bile salt solution for different time intervals, such as 0, 30, 90, and 180 min, plated on Lactobacillus MRS Agar plates, and incubated at 37°C [28–30]. In aspects of control, bacterial cells were suspended only in physiological saline solution without exposure to different bile salt-concentrated solutions and were similarly plated on MRS agar.

  $$\begin{array}{c} \text{Viable count of bacteria in water }\left(\text{CFU}/\text{mL}\right)=\left(\text{no}.\text{of colonies on agar plate}\times \text{dilution factor}\right)/\text{volume of culture plated in mL}. \end{array}$$

#### 2.1.8. Statistical and Data Analysis

Collected data were recorded and analyzed using Microsoft (MS) Excel 2013 version. The outputs were presented in tabular formats, reporting data as mean ± standard deviation. Data regarding the viable count of bacterial colonies after exposing them to pH and bile salt were analyzed by one-way analysis of variance (ANOVA) and post hoc test using Statistical Package for the Social Sciences (SPSS) software, with statistical significance set at *p* < 0.05.

### 2.2. Phase II: In Vivo Aquarium Feeding Trial

#### 2.2.1. Study Area, Duration, and Experimental Setup

The experiment was conducted in aquarium at the Fish Ecophysiology Laboratory, BAU, Mymensingh, over a period of 120 days. A selection of 132 healthy PL, all of similar size and age, were carefully brought and acclimatized in the aquarium. They were kept there for a period of 3 days, being fed with the basal diet thrice a day, and continuous aeration was maintained before starting them with probiotic treatment. The research experiment was conducted using three sets of aquaria (with a capacity of 60 L), each with replication for three treatments. Treatment 1 (T1): fed lab-isolated mixed probiotics (quantified in phase I), treatment 2 (T2): fed commercial probiotics, and control (T0): fed no probiotic in replicate aquaria (thus, six aquaria labeled T1R1, T1R2, T2R1, T2R2, T0R1, and T0R2 were maintained). In each aquarium, 22 prawn PL were stocked with a stocking density of 1 PL per 2.72 L of water. PVC pipe bundles were provided as shelter to reduce cannibalism. All the aquaria were provided with continuous aeration; with temperature regulated using heaters during winter, and about 75% water was exchanged at 10 days interval, and siphoned the uneaten feed out daily. The water quality parameters were monitored regularly, and prepared feeds were fed according to the treatments as described below.

#### 2.2.2. Determination of Water Quality Parameters

The water quality parameters such as temperature, dissolved oxygen (DO), pH, and ammonia concentrations were monitored every week by using a mercury glass thermometer (Saraan, Denmark), DO test kit (Biosol, AA Biotech, India), pH test kit (AQUA-VBC, Thailand) and ammonia test kit (AQUA-VBC, Thailand), respectively as described in as described by Hossain et al. [31].

#### 2.2.3. Preparation of Probiotic Supplemented Feed, Feeding, and Monitoring

A commercial prawn nursery feed (sinking type fine crumble, Mega Feed and Spectra Hexa Feeds Limited, Dhaka, Bangladesh) composed of 11% moisture, 7% crude lipid, 38% crude protein, and 4% crude fiber (extracted from the product label/datasheet as declared by the manufacturer) was used as base for the preparation of probiotic mixed feed. This feed was then mixed with probiotics as follows:

##### 2.2.3.1. Preparation of Lab Isolated Multispecies Probiotic Mixed Feed for T1

Four bacterial strains obtained in phase I, namely *Enterococcus faecium*, *Glutamicibacter mysorens*, *Bacillus cereus*, *and Staphylococcus succinus*, were cultured again in nutrient broth media (at 30°C for a duration of 48 h) and their concentration in the broth culture was measured through serial dilution following standard protocol [32]. The cell concentration was maintained to 10^8^ CFU/g of feed [33, 34]. For this reason, the broth cultures obtained from the refrigerator, appropriate amounts for each culture (each strain) were carefully transferred to Eppendorf tubes and centrifuged (at 5000 rpm for 30 min). The concentrated probiotic mixture was then collected using a pipette and mixed with commercial basal feed, a commercial binder (Nutrigel) was used to facilitate a uniform distribution throughout the feed matrix. Subsequently, the probiotic-integrated feed underwent a drying process under a gentle airflow for a period of 30 min, crucial to maintain the stability of the probiotics-integrated feed. Finally, the feed was stored in a refrigerator at 4°C and used in the growth trial.

##### 2.2.3.2. Preparation of Commercial Probiotic Mixed Feed for T2

A commercial probiotic (Safegut, Eskayef Pharmaceuticals Limited, Dhaka, Bangladesh) was chosen for the Treatment T2, a widely applied product in local prawn and shrimp aquaculture. According to the manufacturer's specifications, it contains lactic acid *Bacillus*, *Bacillus subtilis*, *Bacillus licheniformis*, *Aspergillus oryzae*, *Aspergillus niger*, *Saccharomyces boulardii*, Vitamins, and Enzymes. The available concentration was 3 × 10^9^ CFU/g of powder (Table S1). Farmers commonly use this formulation, which includes a consortium of Bacillus and lactic acid bacteria found to enhance the growth and water quality in aquaculture species [35], making it both a practical and scientifically relevant comparator for evaluating host-derived probiotics. To achieve a concentration of 1 × 10^8^ CFU/g, 1 g of this probiotic powder was added to 30 g of commercial basal feed and mixed uniformly using the commercial binder (Nutrigel) in a similar way as above. The prepared feed was then air-dried briefly and stored in a refrigerator (at 4°C) and used.

For T0 (control) same commercial feed without any probiotics was used. The prawn PL was then fed with experimental diets thrice a day at 10% of their body weight and checked regularly to see whether the feeds were consumed or not.

Prawn PL were sampled (using a scoop net) at every 2 weeks interval. The PL were carefully transferred in a beaker containing little amount of water (allowed them to rest and thus, were not anesthetized during sampling), and their weight was measured carefully using an electric balance. Subsequently, the growth performance was calculated in terms of average weight gain, percent weight gain, and specific growth rate (SGR). Feed conversion ratios (FCRs) and feed conversion efficiencies (FCEs) were also calculated for different treatments.

### 2.3. Statistical Analysis

The raw data were preserved, and necessary analyses were done using the R environment [36]. Homogeneity and normality of the data set were confirmed using the “car” [37] package. One-way ANOVA was performed to test the interaction effect of probiotics on growth performance and water quality parameters, followed by the post hoc test. All the illustrations were created using a package called “ggplot2” [38].

## 3. Results

### 3.1. Isolation and Molecular Identification of Selected Isolates

**Figure 1:**
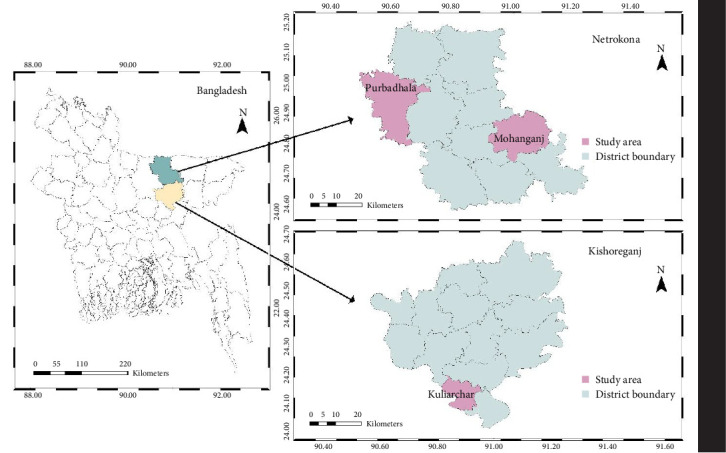
Geographic locations of natural habitats sampled for wild *Macrobrachium rosenbergii*, which were used to isolate and identify potential host-derived probiotic bacteria.

A total of 57 bacterial isolates were collected from 3 sampling stations and preserved as slant cultures. Subsequently, from the entire pool of isolates, a total of nine were chosen, considering the similarities and variations of their colony characteristics for molecular identification and further comprehensive analysis. Among the final set of nine isolates, four were selected from study area 1, 2 from study area 2, and the remaining three from study area 3 (Figure 1).

**Figure 2:**
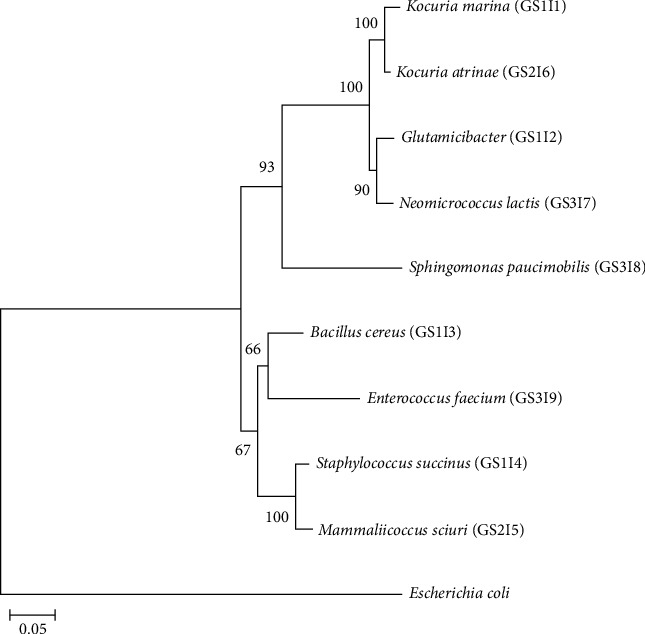
Phylogenetic tree illustrating evolutionary analysis by neighbor-joining method among the isolates (obtained from the gut of *Macrobrachium rosenbergii* to identify potential probiotic bacteria) with an outgroup of *Escherichia coli*. The number at the apex of the clades represents the bootstrap value (out of 100), indicating the confidence level of the branching pattern observed. Scale bar 0.05 reflects a 5% sequence divergence. The letters G, S, and I in the isolate IDs denote, G = Golda (the local name of *M. rosenbergii*), S = study area, and I = isolate number.

Molecular identification of isolates was carried out using Sanger sequencing of the 16s rRNA gene and analyzed using BioEdit software. After comparing the sequence data of the nine selected isolates with NCBI database, with similarities ranging from 98.59% to 100%, they were identified as *Kocuria marina* (GS1I1; [39]), *Glutamicibacter mysorens* (GS1I2; [40]), *Bacillus cereus* (GS1I3; [41]), *Staphylococcus succinus* (GS1I4; [42]), *Mammaliicoccus sciuri* (GS2I5; [43, 44]), *Kocuria atrinae* (GS2I6; [45]), *Neomicrococcus lactis* (GS3I7; [46]), *Sphingomonas paucimobilis* (GS3I8; [47, 48]), and *Enterococcus faecium* (GS3I9; [49]) (Table 1 and Table S2). A comprehensive overview associated with the background information of detected isolates with their sources in nature, and functional properties is summarized, and the phylogenetic tree demonstrates the neighbor-joining evolutionary relationships among identified isolates. *Escherichia coli* represents an out-group, showing the evolutionary distance between it and the identified isolates (Figure 2). This phylogenetic tree was constructed with a scale bar of 0.05, indicating 0.05 nucleotide substitutions per site.

### 3.2. Potentiality Test: pH and Bile Salt Tolerance

Growth responses of the isolates varied under acidic conditions (pH 2, 3, and 4) over 90 min (0, 30, 60, and 90 min). In the control (physiological saline, no pH stress), all isolates grew normally. At pH 2, most isolates showed strong inhibition, with GS3I7 (*Neomicrococcus lactis*) and GS3I8 (*Sphingomonas paucimobilis*) completely losing viability. A few isolates, including GS1I2 (*Glutamicibacter mysorens*) and GS1I4 (*Staphylococcus succinus*), survived only at the initial (0 min) exposure. At pH 3, GS1I4 maintained moderate growth up to 60 min, while GS3I8 showed slight recovery after 30 min. At pH 4, most isolates exhibited improved tolerance, with GS1I3 (*Bacillus cereus*) and GS3I9 (*Enterococcus faecium*) consistently maintaining high viability across all time points. However, even for tolerant isolates, prolonged exposure reduced viable counts; for example, GS1I3 declined from log_10_ 4.44 at 0 min to 4.14 after 90 min at pH 2.

Statistical analysis (one-way ANOVA, Tukey's test) confirmed significant viability loss at pH 2 (*p* < 0.05), moderate tolerance at pH 3, and relatively stable survival at pH 4. *B. cereus* and *E. faecium* showed no significant differences between control and treatments at pH 4, indicating strong acid tolerance (Table 2).

Isolates were exposed to bile salts (0.3%, 0.5%, and 1%) at pH 8 for up to 180 min. In the control (physiological saline), all isolates grew well (log_10_ 4.51–4.72 CFU/mL). At 0.3% bile salts, all isolates maintained high viability without significant differences compared to control. At 0.5%, moderate reductions were observed in several isolates, including GS2I5 (*Mammaliicoccus sciuri*), which declined from log_10_ 4.39 (90 min, 0.3%) to 4.29 (90 min, 0.5%). At 1% bile salts, inhibitory effects were pronounced: GS1I1 (*Kocuria marina*), GS2I6 (*K. atrinae*), GS3I7 (*N. lactis*), and GS3I8 (*S. paucimobilis*) showed complete loss of viability by 180 min. In contrast, GS1I3 (*B. cereus*) and GS3I9 (*E. faecium*) remained viable across all concentrations, with little reduction between 0.3% and 0.5%.

ANOVA results indicated minimal differences at 0.3% (*p*  > 0.05), moderate significance at 0.5%, and highly significant reductions at 1% (*p* < 0.05). The strongest tolerance was again observed in *B. cereus* and *E. faecium*, while sensitive isolates displayed “no growth” at the highest concentration (Table 3).

### 3.3. Changes in Water Quality Parameters

As the study was conducted under controlled conditions for a limited duration, the observed variations reflect treatment effects rather than seasonal or monthly changes. All measured water quality parameters remained within the productive range throughout the trial, with temperature regulated using heaters during winter and DO maintained by aeration. Treatment effects were most evident for pH and ammonia levels.

**Figure 3:**
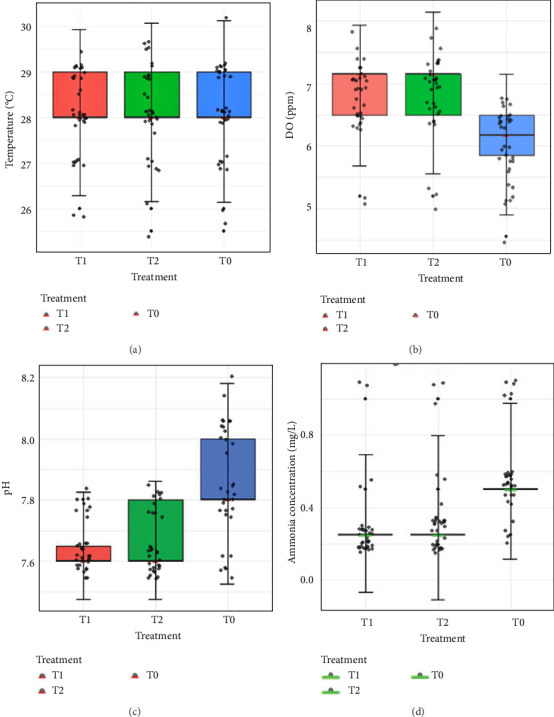
Changes in water quality parameters during *M. rosenbergii* growth trail in different treatments, T1: fed lab-isolated mixed probiotics; T2: fed commercial probiotics, and T0: control fed (no probiotic) over the period of 120 days. The water quality, such as (A) temperature, (B) dissolved oxygen (DO), (C) pH, and (D) ammonia concentration, was monitored weekly basis.

Temperature varied between 25.5 and 30°C, with a stable mean range of 27.5 ± 0.5 to 29 ± 0.5°C across treatments (Figure 3A). DO ranged from 4.55 to 7.8 ppm, averaging 6.2 ± 0.3 to 7.1 ± 2 ppm, and remained within the optimum range for prawn culture throughout the study (Figure 3B).

pH fluctuated between 7.6 and 8.15. In the control (T0), the average was 7.8 ± 0.2, whereas probiotic-treated groups (T1 and T2) maintained slightly lower and more stable values. Notably, pH in T1 remained closer to the optimum range, showing significant differences compared with T0 (*p* < 0.05; Figure 3C). Ammonia concentrations ranged from 0.25 to 1.0 mg/L. Probiotic treatments (T1 and T2) significantly reduced ammonia accumulation relative to T0 (*p* < 0.05). Mean values were 0.36 ± 0.06 mg/L in T0 compared with 0.28 ± 0.03 mg/L in both T1 and T2, with T1 consistently showing the lowest levels (Figure 3D).

Overall, probiotic supplementation—particularly T1—contributed to maintaining more favorable water quality conditions compared with the control.

### 3.4. Growth Performance (Average Weight Gain, Percent Weight Gain, and SGR) and Feed Utilization (FCR and FCE)

Growth responses of *M. rosenbergii* differed among treatments supplemented with laboratory-isolated probiotics (T1), commercial probiotics (T2), and the control (T0). Average weight gain showed a clear improvement in probiotic-supplemented groups. Initial average weights were 0.13 ± 0.002 g (T1), 0.12 ± 0.001 g (T2), and 0.13 ± 0.005 g (T0), increasing to 3.92 ± 0.08 g, 3.17 ± 0.06 g, and 2.31 ± 0.13 g, respectively, after 120 days. While one-way ANOVA across the entire trial showed no overall significance (*p* > 0.05), Tukey's test revealed notable mean differences, particularly between T1 and T0 (1.61 g) and between T2 and T0 (0.86 g) at the end. Importantly, at each 15-day sampling interval, significant differences (*p* < 0.001) in mean weight gain were consistently observed (Table 4 and Table S3).

Percent weight gain (%) followed a similar pattern. Probiotic treatments (T1, T2) maintained significantly higher gains than T0 (Table 4). SGR% also reflected superior performance in probiotic groups. SGR values were 2.87% ± 0.03% (T1), 2.72% ± 0.01% (T2), and 2.36% ± 0.05% (T0). The highest SGR was recorded in T1, which was significantly greater than T0 (*p* < 0.05), though differences between T1 and T2 were not significant (Table 4).

Overall, the growth performance data indicate that the laboratory-isolated probiotic (T1) conferred the greatest benefit, followed by the commercial probiotic (T2), both of which outperformed the control (T0) (Table 4 and Table S3).

Laboratory-isolated probiotics (T1) consistently achieved the lowest FCR (0.32 ± 0.09) and highest FCE (3.15 ± 0.5) compared with the commercial probiotic group (T2; FCR 0.66 ± 0.10, FCE 2.90 ± 0.38) and the control (T0; FCR 1.13 ± 0.14, FCE 1.96 ± 0.23). The control group exhibited the widest fluctuations, while T1 maintained stable performance across sampling intervals. Statistical analysis confirmed that T1 differed significantly (*p* < 0.001) from both T2 and T0 at all-time points, whereas differences between T2 and T0 were generally not significant. These results demonstrate that host-derived probiotics enhanced feed utilization efficiency more effectively than the commercial probiotic or the control (Table 4).

## 4. Discussion

The gastrointestinal tract of *M. rosenbergii* harbored diverse bacteria, from which nine isolates were identified using 16S rRNA sequencing. These included *Bacillus cereus*, *Enterococcus faecium*, *Glutamicibacter mysorens*, *Staphylococcus succinus*, and others (Tables 1 and 2). Both *B. cereus* and *E. faecium* are well-documented probiotics in aquaculture [41, 49–51], while several isolates, such as *Kocuria atrinae*, *Mammaliicoccus sciuri*, and *Neomicrococcus lactis*, represent relatively novel candidates. The recovery of such diversity underscores the potential of host-derived microbiota as a reservoir for probiotic development [52, 53].

The in vitro assays confirmed that acid and bile tolerance varied widely among isolates. While most strains lost viability at pH 2, *B. cereus* and *E. faecium* maintained high survival, consistent with previous reports [41, 54]. *S. succinus* and *G. mysorens* showed moderate viability at pH 3–4 and across bile concentrations, suggesting potential for gut colonization [55, 56]. In contrast, *Kocuria* spp. and *N. lactis* were more sensitive, highlighting the importance of functional screening before probiotic application [46, 57].

Probiotic treatments—particularly the lab-isolated consortium (T1)—stabilized water quality by lowering ammonia and moderating pH fluctuations compared with the control. These improvements are likely due to enhanced microbial activity and nitrification [58, 59], reducing the accumulation of toxic metabolites from uneaten feed. Similar probiotic-mediated effects on water quality have been reported in shrimp and carp systems, supporting the dual role of probiotics in host health and environmental management [35, 60, 61].

Growth responses clearly reflected the probiotic effects. Prawns in T1 exhibited the highest average weight gain, percent weight gain, and SGR, followed by T2 and then T0 (Table 4). Although overall ANOVA sometimes failed to detect differences across the full trial, significant improvements emerged at nearly every sampling point. This suggests that probiotics influence growth trajectories over time, even when endpoint measures appear statistically conservative [5, 62, 63]. Importantly, FCR was lowest and FCE highest in T1, confirming improved feed utilization—consistent with probiotic roles in enzyme production, nutrient absorption, and gut health enhancement [63].

Both probiotic treatments enhanced growth and water quality relative to control, but the lab-isolated consortium (T1) consistently outperformed the commercial product (T2). Similar trends were reported by Ghosh et al. [62], emphasizing the benefits of optimized probiotic formulations in freshwater prawn culture. The superior performance of the host-derived consortium (T1) suggests potential economic advantages over commercial probiotics and may reduce dependency on imported products, though formal economic evaluation remains necessary.

While probiotics are generally considered beneficial in aquaculture, their use may carry potential risks, including the introduction of antibiotic resistance genes, disruption of native microbial communities, or unintended alterations in water quality [64, 65]. The present study emphasizes the advantage of host-derived strains, which may be better adapted to colonize the prawn gut and interact with the host microbiome and did not adversely affect water quality. No disease outbreaks or abnormal mortalities were observed, suggesting that carefully screened, species-derived probiotics can be safely applied for superior outcomes compared to terrestrial or generalized probiotic formulations [66, 67]. Nonetheless, ongoing monitoring and functional assessment are recommended to minimize any potential risks associated with probiotic use. The findings highlight the promise of integrating host-derived probiotics into *M. rosenbergii* culture systems. Beyond improving growth and feed conversion, these probiotics contributed to water quality regulation—an essential factor in intensive aquaculture. The ability of lab-isolated strains to rival or surpass commercial probiotics underscores the potential for developing locally adapted, cost-effective formulations.

## 5. Conclusion

This study established that gut-derived bacteria from *M. rosenbergii* contain promising probiotic candidates. Among the nine isolates identified, *Bacillus cereus* and *Enterococcus faecium* demonstrated the strongest tolerance to acid and bile, while others, such as *Glutamicibacter mysorens* and *Staphylococcus succinus*, displayed moderate potential. In aquarium trials, the laboratory-isolated probiotic consortium (T1) improved water quality by lowering ammonia, enhanced growth performances, showed better feed utilization compared with both the control and a commercial probiotic. These findings emphasize the value of host-derived probiotics as sustainable alternatives to terrestrial or non-native formulations. However, the current study was restricted to short-term trials with a mixed probiotic group; their specific roles were not individually validated. Future investigation should include large-scale pond trials and functional studies (e.g., enzyme production, immune modulation, pathogen resistance) to establish long-term usability. Such work will help confirm host-specific, locally adapted probiotic consortium for improved and sustainable freshwater prawn aquaculture.

## Figures and Tables

**Table 1:** Selected isolates obtained from the *M. rosenbergii* were identified through 16S rRNA sequencing using the Sanger method.

Isolate IDs	Species confirmed	Percent of similarity compared to the NCBI (%)	Primer used	Source	Procedure
^a^GS1I1	*Kocuria marina* [39]	99.00	27F and 1492R [24]	NCBI (National Center for Biotechnology Information)	Using Nucleotide BLAST (Basic Local Alignment Search Tool)
GS1I2	*Glutamicibacter mysorens* [40]	99.78			
GS1I3	*Bacillus cereus* [41]	100			
GS1I4	*Staphylococcus succinus* [42]	100			
GS2I5	*Mammaliicoccus sciuri* [43, 44]	99.03			
GS2I6	*Kocuria atrinae* [45]	99.86			
GS3I7	*Neomicrococcus lactis* [46]	100			
GS3I8	*Sphingomonas paucimobilis* [47, 48]	99.22			
GS3I9	*Enterococcus faecium* [49]	98.59			

*Note:* The sequence data were blasted against the National Center for Biotechnology Information (NCBI) database to obtain percent of similarities.

^a^The letters G, S, and I in the isolate IDs are indicating, G = Golda (which is the local name of *M. rosenbergii*), S = study area, and I = isolate number.

**Table 2:** Tolerance of selected isolates to different levels of pH.

Isolates ID	Control	0 min	30 min	60 min	90 min
Treatment in pH 2 (values are presented as mean ± SEM; *n* = 3)					

^1^GS1I1	^2^4.44 ± 0.02^a^	N/G^b^	N/G^b^	N/G^b^	N/G^b^
GS1I2	4.46 ± 0.05^a^	4.22 ± 0.05^b^	N/G^c^	N/G^c^	N/G^c^
GS1I3	4.56 ± 0.06^a^	4.44 ± 0.04^ab^	4.25 ± 0.03^bc^	4.18 ± 0.06^bc^	4.14 ± 0.09^c^
GS1I4	4.67 ± 0.02^a^	4.65 ± 0.06^a^	N/G^b^	N/G^b^	N/G^b^
GS1I5	4.49 ± 0.04^a^	N/G^b^	N/G^b^	N/G^b^	N/G^b^
GS1I6	4.29 ± 0.08^a^	N/G^b^	N/G^b^	N/G^b^	N/G^b^
GS1I7	4.22 ± 0.05^a^	N/G^b^	N/G^b^	N/G^b^	N/G^b^
GS1I8	4.18 ± 0.06^a^	4.20 ± 0.1^a^	N/G^b^	N/G^b^	N/G^b^
GS1I9	4.57 ± 0.06^a^	4.04 ± 0.04^b^	3.88 ± 0.06^bc^	3.62 ± 0.1^c^	N/G^d^

Treatment in pH 3 (values are presented as mean ± SEM; *n* = 3)					

GS1I1	4.44 ± 0.02^a^	N/G^b^	N/G^b^	N/G^b^	N/G^b^
GS1I2	4.46 ± 0.05^a^	4.18 ± 0.06^b^	4.27 ± 0.1^ab^	N/G^c^	N/G^c^
GS1I3	4.56 ± 0.06^a^	4.57 ± 0.05^a^	4.25 ± 0.03^bc^	4.46 ± 0.05^ab^	4.22 ± 0.05^c^
GS1I4	4.67 ± 0.02^a^	4.63 ± 0.04^a^	4.16 ± 0.03^b^	N/G^c^	N/G^c^
GS1I5	4.49 ± 0.04^a^	4.41 ± 0.02^ab^	4.24 ± 0.07^ab^	4.20 ± 0.1^b^	N/G^c^
GS1I6	4.29 ± 0.08^a^	N/G^b^	N/G^b^	N/G^b^	N/G^b^
GS1I7	4.22 ± 0.05^a^	N/G^b^	N/G^b^	N/G^b^	N/G^b^
GS1I8	4.18 ± 0.06^a^	4.34 ± 0.1^a^	N/G^b^	N/G^b^	N/G^b^
GS1I9	4.57 ± 0.06^a^	4.48 ± 0.03^a^	4.16 ± 0.03^b^	4.07 ± 0.07^b^	3.72 ± 0.1^c^

Treatment in pH 4 (values are presented as mean ± SEM; *n* = 3)					

GS1I1	4.44 ± 0.02^a^	4.27 ± 0.05^b^	N/G^c^	N/G^c^	N/G^c^
GS1I2	4.46 ± 0.05^a^	4.33 ± 0.08^a^	4.04 ± 0.04^b^	N/G^c^	N/G^c^
GS1I3	4.56 ± 0.06	4.62 ± 0.05	4.62 ± 0.03	4.53 ± 0.04	4.48 ± 0.03
GS1I4	4.67 ± 0.02^a^	4.67 ± 0.04^a^	4.46 ± 0.05^b^	N/G^c^	N/G^c^
GS1I5	4.49 ± 0.04^a^	4.42 ± 0.06^a^	4.50 ± 0.05^a^	4.39 ± 0.02^ab^	4.14 ± 0.09^b^
GS1I6	4.29 ± 0.08^a^	3.88 ± 0.06^b^	N/G^c^	N/G^c^	N/G^c^
GS1I7	4.22 ± 0.05^a^	N/G^b^	N/G^b^	N/G^b^	N/G^b^
GS1I8	4.18 ± 0.06^b^	4.44 ± 0.05^a^	4.33 ± 0.08^ab^	4.18 ± 0.06^b^	N/G^c^
GS1I9	4.57 ± 0.06^a^	4.16 ± 0.1^b^	4.46 ± 0.05^ab^	4.56 ± 0.06^a^	4.51 ± 0.02^a^

*Note:* Growth compared to control after treatment at pH 2, 3, and 4 across the time intervals of 0, 30, 60, and 90 min using one-way ANOVA (analysis of variance). The values are presented as means ± SEM, where *n* = 3 per treatment group. Means in each row with a different superscript letter refer to a significant difference (***p* < 0.05**) according to the analysis of one-way ANOVA and the post hoc test.

Abbreviation: N/G, no growth.

^1^The letters G, S, and I in the isolate IDs are indicating, G = Golda (which is the local name of *M. rosenbergii*), S = study area, and I = isolate number.

^2^Bacterial counts are expressed as log_10_ CFU/mL.

**Table 3:** Tolerance of selected isolates to different levels of bile treatments.

Isolates ID	Control	0 min	30 min	90 min	180 min
Treatment in 0.3% bile salt (values are presented as mean ± SEM; *n* = 3)					

^1^GS1I1	^2^4.60 ± 0.04^a^	4.59 ± 0.03^a^	4.48 ± 0.03^a^	4.30 ± 0.04^b^	4.19 ± 0.03^b^
GS1I2	4.65 ± 0.06^a^	4.63 ± 0.05^a^	4.51 ± 0.03^ab^	4.36 ± 0.07^b^	4.07 ± 0.07^c^
GS1I3	4.71 ± 0.009^a^	4.65 ± 0.05^a^	4.69 ± 0.01^a^	4.55 ± 0.05^ab^	4.42 ± 0.06^b^
GS1I4	4.67 ± 0.05^a^	4.64 ± 0.06^ab^	4.61 ± 0.09^ab^	4.51 ± 0.07^ab^	4.34 ± 0.04^b^
GS2I5	4.67 ± 0.02^a^	4.67 ± 0.04^a^	4.45 ± 0.08^b^	4.39 ± 0.02^bc^	4.25 ± 0.03^c^
GS2I6	4.57 ± 0.03^a^	4.54 ± 0.01^a^	4.49 ± 0.03^ab^	4.20 ± 0.1^bc^	4.02 ± 0.1^c^
GS3I7	4.51 ± 0.03^a^	4.44 ± 0.05^ab^	4.26 ± 0.07^bc^	4.16 ± 0.03^cd^	3.94 ± 0.06^d^
GS3I8	4.60 ± 0.03^a^	4.56 ± 0.05^a^	4.50 ± 0.05^a^	4.42 ± 0.03^a^	4.14 ± 0.09^b^
GS3I9	4.72 ± 0.04	4.70 ± 0.04	4.67 ± 0.04	4.61 ± 0.07	4.56 ± 0.06

Treatment in 0.5% bile salt (values are presented as mean ± SEM; *n* = 3)					

GS1I1	4.60 ± 0.04^a^	4.53 ± 0.04^a^	4.27 ± 0.1^ab^	4.14 ± 0.09^b^	4.12 ± 0.06^b^
GS1I2	4.65 ± 0.06^a^	4.57 ± 0.07^a^	4.44 ± 0.05^a^	3.98 ± 0.09^b^	3.88 ± 0.06^b^
GS1I3	4.71 ± 0.009^a^	4.68 ± 0.04^a^	4.6 ± 0.02^ab^	4.55 ± 0.05^ab^	4.44 ± 0.09^b^
GS1I4	4.67 ± 0.05^a^	4.50 ± 0.07^ab^	4.58 ± 0.08^a^	4.45 ± 0.06^ab^	4.25 ± 0.03^b^
GS2I5	4.67 ± 0.02^a^	4.64 ± 0.02^a^	4.32 ± 0.06^b^	4.29 ± 0.07^b^	3.98 ± 0.09^c^
GS2I6	4.57 ± 0.03^a^	4.49 ± 0.03^a^	4.39 ± 0.02^a^	4.04 ± 0.1^b^	4.04 ± 0.04^b^
GS3I7	4.51 ± 0.03^a^	4.30 ± 0.04^b^	4.18 ± 0.06^bc^	4.04 ± 0.04^c^	N/G^d^
GS3I8	4.60 ± 0.03^a^	4.46 ± 0.09^ab^	4.47 ± 0.07^ab^	4.20 ± 0.1^bc^	4.08 ± 0.04^c^
GS3I9	4.72 ± 0.04	4.68 ± 0.04	4.61 ± 0.09	4.59 ± 0.03	4.55 ± 0.04

Treatment in 1% bile salt (values are presented as mean ± SEM; *n* = 3)					

GS1I1	4.60 ± 0.04^a^	4.02 ± 0.1^b^	4.02 ± 0.1^b^	3.94 ± 0.06^b^	N/G^c^
GS1I2	4.65 ± 0.06^a^	4.29 ± 0.07^ab^	3.94 ± 0.06^bc^	3.92 ± 0.1^bc^	3.62 ± 0.1^c^
GS1I3	4.71 ± 0.009^a^	4.56 ± 0.06^ab^	4.52 ± 0.05^ab^	4.44 ± 0.02^b^	4.35 ± 0.09^b^
GS1I4	4.67 ± 0.05^a^	4.57 ± 0.03^a^	4.47 ± 0.06^a^	4.42 ± 0.09^a^	4.07 ± 0.07^b^
GS2I5	4.67 ± 0.02^a^	4.60 ± 0.06^ab^	4.42 ± 0.06^ab^	4.16 ± 0.2^bc^	3.72 ± 0.1^c^
GS2I6	4.57 ± 0.03^a^	4.40 ± 0.05^ab^	4.23 ± 0.2^ab^	4.04 ± 0.04^b^	N/G^c^
GS3I7	4.51 ± 0.03^a^	4.15 ± 0.07^ab^	4.08 ± 0.04^b^	3.88 ± 0.06^bc^	3.68 ± 0.2^c^
GS3I8	4.60 ± 0.03^a^	4.55 ± 0.09^a^	4.32 ± 0.02^a^	4.24 ± 0.07^a^	3.78 ± 0.1^b^
GS3I9	4.72 ± 0.04^a^	4.61 ± 0.07^ab^	4.60 ± 0.03^ab^	4.49 ± 0.04^b^	4.44 ± 0.05^b^

*Note:* Growth compared to control after treatment at Bile salt concentration 0.3%, 0.5%, and 1% across the time intervals of 0, 30, 90, 180 min using one-way ANOVA (analysis of variance). The values are presented as means ± SEM, where *n* = 3 per treatment group. Means in each row with a different superscript letter refer to a significant difference (***p* < 0.05**) according to the analysis of one-way ANOVA and the post hoc test.

Abbreviation: N/G, no growth.

^1^The letters G, S, and I in the isolate IDs are indicating, G = Golda (which is the local name of *M. rosenbergii*), S = study area, and I = isolate number.

^2^Bacterial counts are expressed as log_10_ CFU/mL.

**Table 4:** Average weight gain (g), percent weight gain (%), and specific growth rate of *M. rosenbergii* among the different treatments; T1: fed lab-isolated mixed probiotics, T2: fed commercial probiotics, and T0: control (fed no probiotic), used to observe the effects of probiotics in aquaria.

Treatment	Initial average body weight (g)	Final average body weight (g)	Average weight gain (g)	Percent weight gain (%)	Specific growth rate (SGR)	Feed conversion ratio (FCR)	Feed conversion efficiency (FCE)
T1	0.13 ± 0.002	3.92 ± 0.08^a^	3.79 ± 0.08^a^	3012.90 ± 112.90^a^	2.87 ± 0.03^a^	0.32 ± 0.09^c^	3.15 ± 0.5^a^
T2	0.12 ± 0.001	3.17 ± 0.06^b^	3.05 ± 0.06^b^	2521.27 ± 26.27^b^	2.72 ± 0.01^a^	0.66 ± 0.10^b^	2.90 ± 0.38
T0	0.13 ± 0.005	2.31 ± 0.13^c^	2.19 ± 0.13^c^	1746.79 ± 30.13^c^	2.36 ± 0.05^b^	1.13 ± 0.14^a^	1.96 ± 0.23^c^
*p*-Value	0.204	~ 0.001	~ 0.001	~ 0.001	~ 0.001	~ 0.001	0.032
Sig. level	NS	*⁣* ^*∗∗*^	*⁣* ^*∗∗*^	*⁣* ^*∗∗*^	*⁣* ^*∗∗*^	*⁣* ^*∗∗*^	*⁣* ^*∗*^

*Note:* The values are presented as means ± SEM (standard error of the mean), means in each column with a different superscript letter refer to a significant difference (*p* < 0.001) according to the analysis of one-way ANOVA (analysis of variance) and the post hoc test.

Abbreviation: NS, not significance.

*⁣* ^*∗*^Significant at 5% level.

*⁣* ^*∗∗*^Significant at 1% level.

## Data Availability

Data will be made available upon request.

## Supporting Information

Supporting Information Table S1: Specification of the commercial probiotic used in the feeding Treatment 2 (T2) to compare the efficacy of lab-isolated probiotics in *M. rosenbergii* over a period of 120 days in aquaria. Table S2: Comprehensive overview of the background information, sources in nature, and functional properties of selected isolates obtained from the *M. rosenbergii* as potential probiotics. Table S3: Statistical analyses of the average weight gain of *M. rosenbergii* among the different treatments; T1: fed lab-isolated mixed probiotics, T2: fed commercial probiotics, and T0: control fed (no probiotic), used to compare the efficacy of lab-isolated probiotics in *M. rosenbergii* over a period of 120 days in aquaria.
