# Supplementary material for: Isolation and Validation of Host-Derived Probiotics From the Giant Freshwater Prawn (Macrobrachium rosenbergii): Impacts on Water Quality and Growth Performance
Source: Aquac Nutr. 2025 Nov 26;2025:9156848. doi: 10.1155/anu/9156848 (PMC12674871; doi:10.1155/anu/9156848)
Supplement: Supporting Information — Table S1: Specification of the commercial probiotic used in the feeding Treatment 2 (T2) to compare the efficacy of lab-isolated probiotics in M. rosenbergii over a period of 120 days in aquaria. Table S2: Comprehensive overview of the background information, sources in nature, and functional properties of selected isolates obtained from the M. rosenbergii as potential probiotics. Table S3: Statistical analyses of the average weight gain of M. rosenbergii among the different treatments; T1: fed lab-isolated mixed probiotics, T2: fed commercial probiotics, and T0: control fed (no probiotic), used to compare the efficacy of lab-isolated probiotics in M. rosenbergii over a period of 120 days in aquaria. [file 9156848.f1.docx]

**Supplementary Table 1:** Specification of the commercial probiotic used in the feeding Treatment 2 (T2) to compare the efficacy of lab-isolated probiotics in *M. rosenbergii* over a period of 120 days in aquaria.

| Product name | Manufacturer | Reported strains and composition* | Declared concentration | Formulation | Application rate |
| --- | --- | --- | --- | --- | --- |
| Safegut | Eskayef Pharmaceuticals Limited, Dhaka, Bangladesh | Lactic acid *Bacillus*, *Bacillus subtilis*, *Bacillus licheniformis*, *Aspergillus oryzae*, *Aspergillus niger*, *Saccharomyces boulardii*, Vitamins, and Enzymes | 3×10^9^ colony forming unit (CFU)/g | Powder | Not mentioned in the label |

*Information as declared on the product label/datasheet. Strain-wise concentration details are not disclosed by the manufacturer.

**Supplementary Table 2:** Comprehensive overview of the background information, sources in nature and functional properties of selected isolates obtained from the *M. rosenbergii* as potential probiotics.

| **Isolate**  **IDs** | **Species identified** | **Gram** | **Shape** | **Source in nature** | **Metabolism/**  **Functional properties** | **References** |
| --- | --- | --- | --- | --- | --- | --- |
| **†**GS1I1 | *Kocuria marina* | Gram (+) | Cocci | skin, soil, and fresh water | aerobic metabolism, possesses catalase activity, and expresses β-galactosidase enzymes | (Kim et al., 2004) |
| GS1I2 | *Glutamicibacter mysorens* | Gram (+) | Cocci | Soil, Mangrove sediment, intestine of marine and freshwater fish | Antiproliferative activity of bioactive compounds and antimicrobial peptides , catalase-positive | (Karthik & Kalyani, 2021) |
| GS1I3 | *Bacillus cereus* | Gram (+) | Bacilli | soil and seawater, plant tissues, , Mammalian and arthropod guts | facultative anaerobe, catabolizes glucose, Show Catalase Positive, oxidase-negative reaction | (Caro-Astorga et al., 2020) |
| GS1I4 | *Staphylococcus succinus* | Gram (+) | Cocci | Upper gastrointestinal tract | Antagonistic activity against uropathogens and proteolytic activity | (Khusro et al., 2018) |
| GS2I5 | *Mammaliicoccus sciuri* | Gram (+) | Cocci | As a novel species isolated from raw Goat milk | coagulase-negative, Catalase-positive with variable oxidase  Activities have appeared | (Madhaiyan et al., 2020);  (Naqqash et al., 2022) |
| GS2I6 | *Kocuria atrinae* | Gram (+) | Cocci | milk of water deer and reindeer | Show Catalase-positive and oxidase-negative reaction, Reduces nitrate to nitrite | (Park et al., 2009) |
| GS3I7 | *Neomicrococcus lactis* | Gram (+) | Cocci | Waste of Milk treatment plant, air, activated sludge, roots of plants | catalase, gelatinase, acid phosphatase positive and oxidase, urease negative | (Prakash et al., 2015) |
| GS3I8 | *Sphingomonas paucimobilis* | Gram (-) | Bacilli | fish skin mucus, soil and water | oxidase and catalase positive, non fermentative, Consume organic compound | (Goker et al., 2017);  (Boutin et al., 2013) |
| GS3I9 | *Enterococcus faecium* | Gram (+) | Cocci | gastrointestinal tract of humans and animals | Lactic acid producing, participate in Citrate, Carbohydrate, Sugar metabolism | (Ramsey et al., 2014) |

**†** The letters G, S and I in the isolate IDs are indicating, G= Golda (which is the local name of *M. rosenbergii*), S = study area and I =isolate number.

**Supplementary Table 3:** Statistical analyses of the average weight gain of *M. rosenbergii* among the different treatments; T1: fed lab-isolated mixed probiotics, T2: fed commercial probiotics and T0: control fed (no probiotic), used to compare the efficacy of lab-isolated probiotics in *M. rosenbergii* over a period of 120 days in aquaria.

|  | Day 0 | Day 15 | Day 30 | Day 45 | Day 60 | Day 75 | Day 90 | Day 105 | Day 120 |
| --- | --- | --- | --- | --- | --- | --- | --- | --- | --- |
| T1 | 0.13 | 0.29a | 0.53a | 0.84a | 1.25a | 1.77a | 2.38a | 3.05a | 3.92a |
| T2 | 0.12 | 0.21b | 0.42b | 0.70b | 0.95b | 1.44b | 2.00a | 2.55 | 3.17b |
| T0 | 0.13 | 0.19c | 0.30c | 0.49c | 0.76c | 1.10c | 1.55b | 1.99c | 2.31c |
| *P* value | 0.204 | ~0.001 | ~0.001 | ~0.001 | ~0.001 | ~0.001 | ~0.001 | ~0.001 | ~0.001 |
| Sig. Level | NS | ** | ** | ** | ** | ** | ** | ** | ** |
|  |  |  |  |  |  |  |  |  |  |
| SEMs | 0.002 | 0.013 | 0.021 | 0.036 | 0.049 | 0.036 | 0.031 | 0.041 | 0.034 |
|  | 0.001 | 0.021 | 0.027 | 0.008 | 0.008 | 0.016 | 0.030 | 0.023 | 0.027 |
|  | 0.005 | 0.012 | 0.017 | 0.031 | 0.038 | 0.051 | 0.030 | 0.023 | 0.030 |

NS = Not significance; * Significant at 5% level; **Significant at 1% level. Data represent the mean values (standard error of the means, SEMs are shown separately) of the treatments. In every sampling date, the *P* value was recorded among the treatments. In a row, values with superscript letters or without letter do not differ significantly whereas values with dissimilar letter differ significantly (as per DMRT, Duncan's Multiple Range Test).
